# A Novel Kinase Inhibitor AX-0085 Inhibits Interferon-γ-Mediated Induction of PD-L1 Expression and Promotes Immune Reaction to Lung Adenocarcinoma Cells

**DOI:** 10.3390/cells11010019

**Published:** 2021-12-22

**Authors:** Jusong Kim, Haeyeon Jang, Gyu Jin Lee, Yelim Hur, Juhee Keum, Jung Ki Jo, Si-Eun Yun, Sung Jun Park, Young Jun Park, Myeong Jun Choi, Kye-Seong Kim, Jaesang Kim

**Affiliations:** 1 Department of Life Science, Ewha Womans University, Seoul 03760, Korea; jusong0425@naver.com (J.K.); yeon7780@naver.com (H.J.); m6mwww12@naver.com (G.J.L.); yarimu@gmail.com (Y.H.); selly1818@hanmail.net (J.K.); 2 Ewha Research Center for Systems Biology, Ewha Womans University, Seoul 03760, Korea; 3 Department of Urology, College of Medicine, Hanyang University, Seoul 04763, Korea; victorjo38@gmail.com; 4 R&D Center, Axceso Biopharma Co., Ltd., Yongin 14056, Korea; seyun@axcesobiopharma.com (S.-E.Y.); sjpark@axcesobiopharma.com (S.J.P.); yjpark@axcesobiopharma.com (Y.J.P.); myeongjun@gmail.com (M.J.C.); 5 Graduate School of Biomedical Science and Engineering, Hanyang University, Seoul 04763, Korea; ks66kim@hanyang.ac.kr; 6 Hanyang Biomedical Research Institute, College of Medicine, Hanyang University, Seoul 04763, Korea

**Keywords:** AX-0085, PD-L1, immune checkpoint, cancer immunotherapy, lung adenocarcinoma

## Abstract

In this study, we describe a novel kinase inhibitor AX-0085 which can suppress the induction of PD-L1 expression by Interferon-γ (IFN-γ) in lung adenocarcinoma (LUAD) cells. AX-0085 effectively blocks JAK2/STAT1 signaling initiated by IFN-γ treatment and prevents nuclear localization of STAT1. Importantly, we demonstrate that AX-0085 reverses the IFN-γ-mediated repression of T cell activation in vitro and enhances the anti-tumor activity of anti-PD-1 antibody in vivo when used in combination. Finally, transcriptomic analyses indicated that AX-0085 is highly specific in targeting the IFN-γ-pathway, thereby raising the possibility of applying this reagent in combination therapy with checkpoint inhibitor antibodies. It may be particularly relevant in cases in which PD-L1-mediated T cell exhaustion leads to immunoevasive phenotypes.

## 1. Introduction

Cancer immunotherapy, in particular via immune checkpoint inhibition, currently represents the most promising therapeutic intervention against multiple types of cancer, including lung cancer, one of the top-ranked cancers in incidence as well as in mortality [1,2,3,4]. Significant improvements in the response rates and/or 5-year overall survival rates compared to traditional chemotherapy have been reported for multiple checkpoint inhibitors targeting CTLA4, PD-1 or PD-L1 [1,5,6,7,8,9]. Unfortunately, the majority of patients do not benefit at all from immune checkpoint blockade indicating an innate resistance mechanism at work [10]. In addition, a significant proportion of patients showing initial response ultimately acquires secondary resistance and succumbs to the disease [11]. The success rate in terms of long-term disease-free survival is still around 30% for non-small cell lung carcinoma, the major subtype accounting for over 85% of LUAD.

Identifying the diverse factors that affect the immune-evasive behaviors of tumor cells and predicting the ultimate outcome of a given immunotherapy are major topics of current investigations. The primary driver mutation, overall tumor mutational burden and tumor microenvironment all seem to be important factors, and efforts to target contributing elements, such as combining mutation-specific targeted therapy with immune checkpoint inhibition, are underway [2,12]. An important interaction between immune cells and tumor cells is mediated by IFN-γ-signaling [13]. This pleiotropic cytokine has been known to act in both ‘anti-tumor’ and ‘pro-tumor’ manners, depending on the types of cancer and contexts of interaction with immune cells [13]. In terms of its tumor-promoting activity, the best-known mechanism involves promotion of expression of immune checkpoint genes, including *PD-L1*. The interaction between PD-L1 and PD-1 expressed, respectively, in tumor cells and in immune cells constitutes the essence of immune checkpoint blockade response [3]. Specifically, the interaction between the two molecules induces T cell exhaustion and inhibits T cell activation required for effective targeting of tumor cells [14,15,16]. Perhaps not surprisingly, monoclonal antibodies targeting PD-1 or PD-L1 are the major tools of immune checkpoint inhibition [1,3]. 

Given the limited success rates, efforts to improve the success of immunotherapy by combining with other treatment modalities have been made extensively [17,18]. One strategy would be to identify chemical reagents to augment the antibody-based therapeutics and abrogate immune checkpoint blockade. Here, we describe the activity of a novel kinase inhibitor AX-0085 which blocks the IFN-γ-induced activation of JAK2/STAT1 pathway and up-regulation of *PD-L1*. We provide evidence in vitro and in vivo that AX-0085 represents a potential therapeutic reagent that can enhance the anti-tumor efficacy of antibody treatments.

## 2. Materials and Methods

### 2.1. Derivation of AX-0085

Multiple derivatives of N-(4-methyl-3-(8-methyl-7-oxo-2-(phenylamino)-7,8-dihydropyrido[2,3-d]pyrimidin-6-yl)phenyl)-3-(trifluoromethyl)benzamide, a type-2 kinase inhibitor interacting with the DFG-out conformation of the spleen tyrosine kinase (Syk) have been synthesized and screened by luciferase assay [19]. Further details on the molecular structure and synthesis of the tested derivatives are available upon request. The promoter region of human PD-L1 (−838 to +114) was inserted between KpnI and XhoI sites of pGL4.14-[luc2/Hygro] vector (Promega, Madison, WI, USA). After transient transfection of reporter plasmids to A549 cells using Lipofectamine 2000 (Invitrogen, Carlsbad, CA, USA), cells were treated with IFN-γ (20 ng/mL) and varying concentrations (0, 0.1, 0.5, and 1.0 μM) of inhibitors. After 24 h, cells were harvested, and luciferase activity was measured using the Luciferase Assay System (Promega).

### 2.2. Cell Culture and Reagents

Human non-small cell lung cancer cell lines A549, HCC827, H226, H358, H460, and H1975 and murine Lewis lung carcinoma cells were purchased from the American Type Culture Collection (ATCC; Manassas, VA, USA). Cells were typically cultured in RPMI-1640 (WELGENE, Gyeongsan, Korea) supplemented with 10% fetal bovine serum (Gibco, Carlsbad, CA, USA) and 1% penicillin/streptomycin (Gibco) in humidified incubators at 37 °C with 5% CO_2_. Recombinant human IFN-γ protein (R&D systems, Minneapolis, MN, USA) was applied at 20 ng/mL, unless indicated otherwise.

### 2.3. Quantitative Real Time PCR

Total RNA from cells was extracted using TRIzol^®^ Reagent (Ambion, Austin, TX, USA), and cDNA was synthesized from 1 µg of total RNA using ImProm-II™ reverse transcriptase (Promega) according to the manufacturers’ protocols. Approximately 10 ng of cDNA was subjected to PCR amplification using SYBR Select Master Mix (Applied Biosystems, Waltham, MA, USA) or KAPR Probe Fast qPCR master mix (KAPA Biosystems, Wilmington, MA, USA) on a CFX96 Real-time PCR detection system (Bio-Rad, Hercules, CA, USA) or Applied Biosystems7300 Real-Time PCR System (Applied Biosystems). The following genes with oligonucleotide primer sequences in parentheses are amplified by PCR: human *PD-L1* (Forward:5′-CCTACTGGCATTTGCTGAACGCAT-3′, Reverse: 5′-ACCATAGCTGATCATGCAGCGGTA-3′); human *ACTB* (Forward 5′- ACAGAGCCTCGCCTTTGC-3, Reverse: 5′- GAAGCCGGCCTTGCACA-3′); human *HPRT1* (Forward: 5′- ACACTGGCAAAACAATGCAG-3′, Reverse: 5′-GTGGGGTCCTTTTCACCAG-3′); human *RPL13A* (5′-GCGGCTGCCGAAGATGG-3′, Reverse: 5′-GGCCCAGCAGTACCTGTT-3′); mouse *PD-L1* (Forward: 5′-GCATTATATTCACAGCCTGC, 3′-CCCTTCAAAAGCTGGTCCTT-3′); mouse *18s ribosomal RNA* (*18s*) (Forward: 5-AGGAATTGACGGAAGGGCACC-3′, Reverse: 5′-GTGCAGCCCCGGACATCTAAG-3′).

### 2.4. Immunoblotting Assay

Cells were lysed with cold RIPA buffer (50 mM Tris-Cl, PH 8.0, 2 mM EDTA, 150 mM NaCl, 1% NP-40, 0.5% Na-Deoxycholate, 0.1% SDS, and 10 mM NaF) supplemented with a mixture of protease inhibitors (Sigma, St. Louis, MO, USA; P8340) and phosphatase inhibitors (Sigma; P5726). The concentration of total protein was measured using BCA protein assay kit (Thermo Scientific Pierce, Rockford, IL, USA). Proteins were resolved by SDS-PAGE and electrotransferred to a PVDF membrane. Primary antibodies used in immunoblot analyses are as follows: anti-hPD-L1 (Abcam, Cambridge, MA, USA; ab205921), anti-Jak2 (Cell Signaling Technology, Beverly, MA, USA; 3230), anti-phospho-Jak2(Cell Signaling Technology; 3771), anti-Stat1 (Cell Signaling Technology; 9172), anti-phospho-Stat1 (Cell Signaling Technology; 9167), anti-mouse PD-L1 (R&D systems; AF1019), anti-β-actin (Santa Cruz Biotechnology, Dallas, TX, USA; sc-47778), and anti-α-tubulin (Sigma; SAB3501072). After applying appropriate secondary antibodies, proteins were detected by enhanced chemiluminescence detection kit (AbClon, Seoul, Korea) and ChemiDoc™ Imaging system (Bio-Rad). Densitometric analyses of immunoblotting results were carried out using ImageJ software (v. 1.8.0). Raw data are provided in Supplementary File S1.

### 2.5. Immunofluorescence Assay

Cells were seeded on four-well slides for 24 h and pre-treated with AX-0085 (0.5 μM) for 2 h before treatment with IFN-γ for an hour. Cells were fixed with 4% paraformaldehyde in PBS for 20 min at 37 °C, permeabilized with 0.2% TritonX-100 for 5 min, and blocked with 3% BSA for 20 min. After incubation with the primary antibody against STAT1 (Santa Cruz Biotechnology, sc-464) overnight at 4 °C, followed by incubation for 30 min with Alexa-594 conjugated goat anti-mouse secondary antibody (Invitrogen; A-11005), cells were subsequently counterstained with 4′6-diamidino-2-phenylindole (DAPI) and examined by LSM 880 confocal laser scanning microscope (Carl Zeiss, Jena, Germany). Densitometric analyses of immunofluorescence assay results were carried out using ImageJ software. Raw data are provided in Supplementary File S1.

### 2.6. Co-Culture of LUAD Cells with PBMC

A total of 2 × 10^5^ A549, HCC827 cells were seeded per a well. After 24 h, cells were treated with cytokine IFN-γ (20 ng/mL) for the first 24 h and with further addition of AX-0085 (0.5 μM) for another 24 h. 2 × 10^5^ peripheral blood mononuclear cells (PBMC; ATCC) were separately cultured and stimulated for 24 h with CD3/CD28 Dynabeads (Gibco, 11161D). Tumor cells and PBMC were co-cultured in 1:1 ratio. After 72 h of co-culture, human IL-2 level in the culture medium was measured using the ELISA kit (Invitrogen, BMS221).

### 2.7. Mouse Xenograft Model

An inoculum of 3 × 10^5^ LLC lung carcinoma cells was injected subcutaneously on the flank of C57BL/6 mice (Orient Bio Inc., Seongnam, Korea) in 100 µL serum-free media. Seven days after injection, the treatment was initiated with intraperitoneal injection of anti-mouse PD-1 antibody (Bioxcell, Lebanon, NH, USA, BE0146) at 400 µg per dose every third day and daily intraperitoneal injection of AX-0085 at 10 mg/kg. The tumor volume was calculated as 0.5 × length × width^2^. The tumors were allowed to grow for 10 days, and mice were sacrificed after 9 days of treatment, and tumor tissues were excised and weighed. The plan for this study was reviewed and approved by the Institution of Animal Care and Use Committee (IACUC) of Ewha Womans University (approval number: IACUC-21-005).

### 2.8. Transcriptome Analysis

RNA was extracted from variably treated A549 cells, and mRNA libraries were prepared using the TruSeq Stranded mRNA Preparation kit (Illumina, San Diego, CA, USA) according to the manufacturer’s instructions. RNA sequencing (RNA-Seq) was performed with Illumina HiSeq2500 sequencing platform for 101-mer paired-end reads. TrimGalore (version 0.6.7) was used for trimming adapter sequences and discarding low-quality reads. The reads were mapped against the human reference genome (GRCh38/hg38) using the STAR alignment program (version 2.7.9a) [20]. Gene expression matrix was generated using FeatureCount function in Rsubread version 2.6.4 [21]. Differentially expressed genes (DEGs) were identified using DESeq2 with the adjusted *p*-value < 0.05 and |fold change| > 2 threshold [22]. Gene set analyses of DEGs for pathways and gene ontology (GO) terms and Gene Set Enrichment Analysis (GSEA) were performed using clusterProfiler version 4.0.4, an R package for interpretation of omics data [23]. The RNAseq data have been deposited in the Gene Expression Omnibus (GEO) database [GEO: GSE185306].

## 3. Results

### 3.1. Derivation of AX-0085 as an Inhibitor of PD-L1 Expression

**Figure 1:**
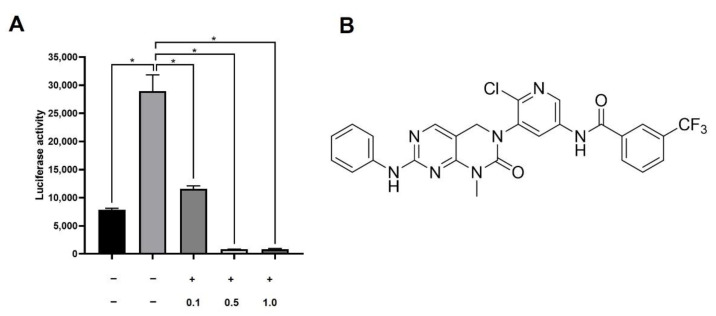
Derivation and structure of AX-0085. (**A**) Results from luciferase-based screen identifying AX-0085. Data are average of two independent experiments and error bars represent standard deviation (* *p*-value of < 0.05 from Student’s *t*-test). (**B**) Molecular structure of AX-0085.

We have designed and synthesized multiple kinase inhibitor candidates expected to inhibit ATP binding and stabilize of DFG-out conformation [19]. They were tested for inhibition of *PD-L1* reporter activation by IFN-γ. The luciferase reporter contained -838 to +114 region of human *PD-L1* (Gene ID: 29126) and showed over three-fold activation by IFN-γ when tested in A549 cells. (Figure 1A). Inhibition was tested at three different concentrations, 0.1 μM, 0.5 μM, and 1.0 μM for each candidate inhibitors. AX-0085 was the sole chemical that showed near saturation level inhibition at 0.5 μM and was chosen for subsequent analyses (Figure 1A,B).

### 3.2. AX-0085 Inhibits Induction of PD-L1 by IFN-γ

**Figure 2:**
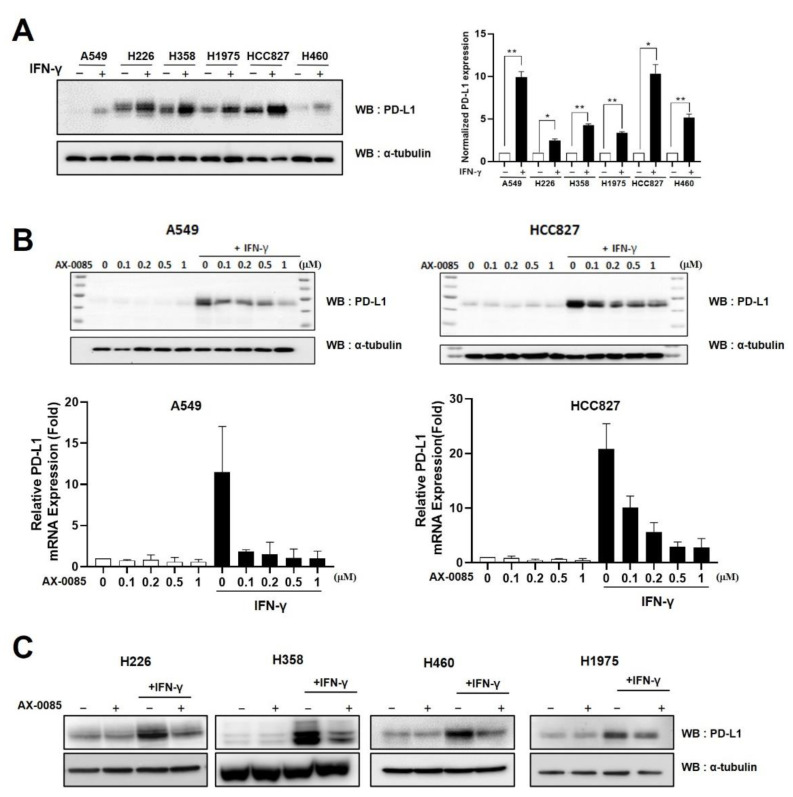
AX-0085 inhibits IFN-γ-induced PD-L1 expression. (**A**) Immunoblots showing up-regulation of PD-L1 expression with the addition of IFN-γ at 20 ng/mL for 24 h. LUAD cell lines used are indicated. ɑ–tubulin was used as the loading control. The graph on the right shows the results from the quantitative real time RT-PCR assay. Data are mean ± SEM of three independent experiments. Statistical significance is indicated (* *p*-value of <0.05, ** *p*-value of <0.01 from Student’s *t*-test). (**B**) Immunoblot showing inhibition of IFN-γ-induced PD-L1 up-regulation by AX-0085 in a concentration-dependent manner in A549 and HCC827 cells. Cells were treated with 20 ng/mL of IFN-γ for 24 h prior to application of indicated levels of AX-0085 for another 24 h. ɑ–tubulin was used as the loading control. Graphs at the bottom show results from the quantitative real time RT-PCR. Data are mean ± SEM of three independent experiments. Statistical significance is indicated (* *p*-value of <0.05, ** *p*-value of <0.01 from Student’s *t*-test). (**C**) Immunoblots showing changes in PD-L1 expression induced by sequential addition of IFN-γ (20 ng/mL) and AX-0085 (0.5 μM) as in (**B**). LUAD cell lines used are indicated. ɑ–tubulin was used as the loading control.

We examined the effect of IFN-γ treatment on PD-L1 expression in LUAD cells. The basal levels of PD-L1 expression varied among the six cell lines we tested, but all showed significant up-regulation upon IFN-γ treatment at both the protein and mRNA levels (Figure 2A). We selected A549 with a relatively low basal expression level and HCC827 with a relatively high basal expression level for detailed analyses. We first tested if AX-0085 inhibited IFN-γ-induced up-regulation of PD-L1. Indeed, AX-0085 showed a concentration-dependent inhibition of PD-L1 induction, typically reaching the maximal effect around 0.5 μM similar to the *PD-L1* reporter (Figure 2B). The inhibition was seen for both A549 and HCC827 cells and for both protein and mRNA levels. The inhibitory effect was also seen in the other four LUAD cell lines (Figure 2C).

**Figure 3:**
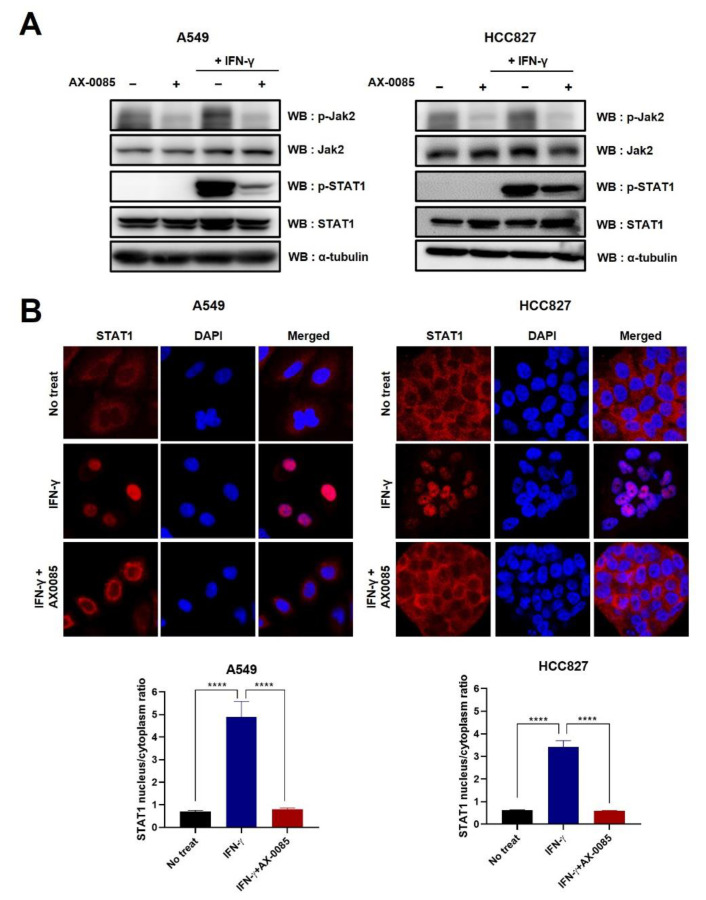
AX-0085 targets the JAK2-STAT1 signaling pathway. (**A**) Immunoblots showing inhibition of IFN-γ-induced phosphorylation of JAK2 and STAT1 by AX-0085. A549 cells and HCC827 cells were treated for 2 h with AX-0085 (0.5 μM) prior to treatment with IFN-γ 20 ng/mL for 1 h. Antibodies specific to phosphorylated forms of JAK2 (p-JAK2) and STAT1 (p-STAT1) and antibodies for the total JAK2 and STAT1 proteins were used. ɑ–tubulin was used as the loading control. (**B**) Immunocytochemical staining showing inhibition of IFN-γ-induced nuclear localization of STAT1 by AX-0085. DAPI counterstain visualizes nuclei. Graphs below show nucleocytoplasmic ratios of anti-STAT1 immunofluorescence. For each condition, 10 A549 cells and 16 HCC827 cells were examined. Statistical significance is indicated (**** *p*-value of <0.0001 from Student’s *t*-test).

We next examined if AX-0085 blocked IFN-γ-induced activation of the JAK2/STAT1 pathway. For both A549 and HCC827 cells, pre-treatment of AX-0085 effectively reduced IFN-γ-induced phosphorylation of JAK2 and STAT1 in the absence of change in their total protein levels (Figure 3A). STAT1 is known to be present in cytoplasm in an inactive and unphosphorylated state but localize to nucleus upon phosphorylation and function in transcriptional activation of target genes. Upon IFN-γ treatment, nuclear localization of STAT1 was readily observed in both A549 and HCC827 cells (Figure 3B). Consistent with the inhibition of phosphorylation of STAT1, AX-0085 effectively blocked nuclear localization of STAT1, indicating that AX-0085 works at the level of JAK2/STAT1 activation by IFN-γ (Figure 3B).

### 3.3. Transcriptomic Analysis of AX-0085 Activity Using A549 Cells

**Figure 4:**
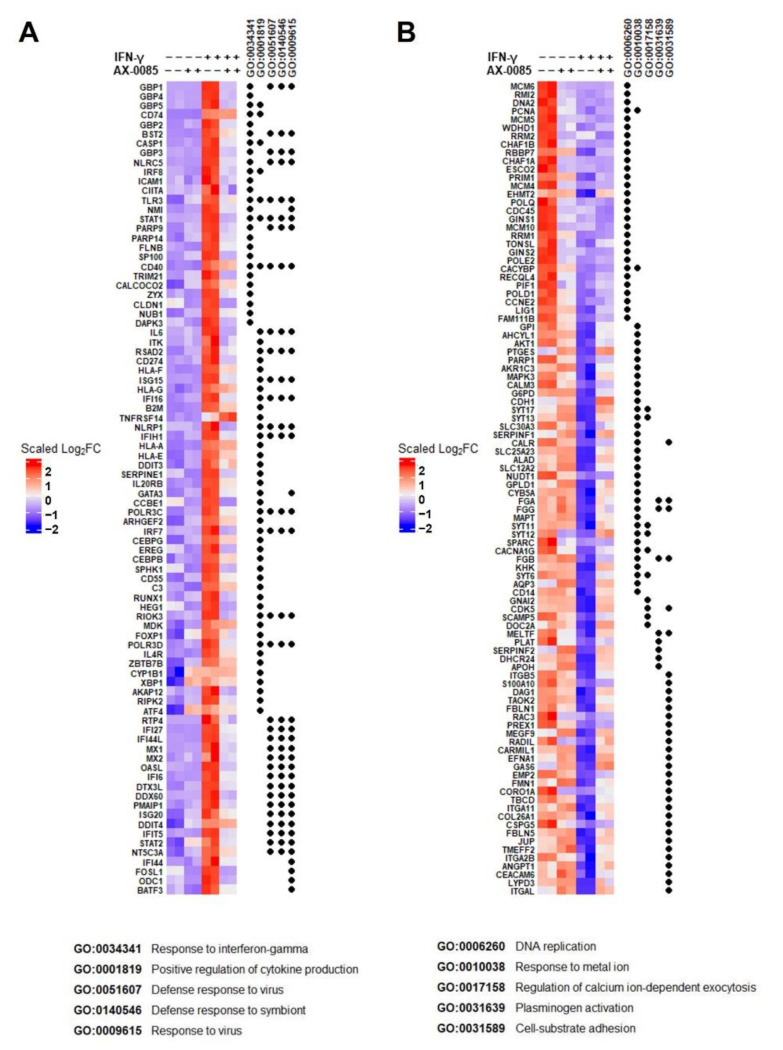
Transcriptomic analyses of IFN-γ signaling by AX-0085. Heat map representation of gene expression. Genes from the top 5 up-regulated (**A**) and down-regulated (**B**) GO term groups upon IFN-γ treatment are shown on the left in duplicates. The changes in their expression upon further treatment with AX-0085 are shown on the right. For GO IDs specified above, the groupings are indicated by black dots and terms are listed at the bottom (also listed in Supplementary Files S2 and S3).

In order to further understand the detailed activity of AX-0085, we carried out gene expression profiling for A549 cells treated with IFN-γ and AX-0085. Duplicate samples of cells that are untreated, IFN-γ-treated, AX-0085-treated, or combinatorially treated with both IFN-γ and AX-0085 treated were prepared and subjected to RNAseq analyses. We focused on the gene expression changes upon IFN-γ-treatment and on the alterations to the pattern by further addition of AX-0085. Significantly up-regulated and down-regulated genes from IFN-γ-treatment alone were subjected to gene set analyses. Most notably, top up-regulated GO terms included ‘response to interferon-gamma ‘, ‘positive regulation of cytokine production’, ‘defense response to virus’, ‘defense response to symbiont’, and ‘response to virus’. Figure 4 and Supplementary FileSupplementary File S2 consistent with response to IFN-γ. Importantly, most of the genes belonging to these GO term groups showed clear down-regulation upon further addition of AX-0085 (Figure 4A). Most of the genes belonging to the top down-regulated GO term groups showed up-regulation by further addition of AX-0085, although ‘DNA replication’ group genes were mostly non-responsive to AX-0085 (Figure 4B and Supplementary FileSupplementary File S3).

### 3.4. Activation of T Cell by AX-0085

**Figure 5:**
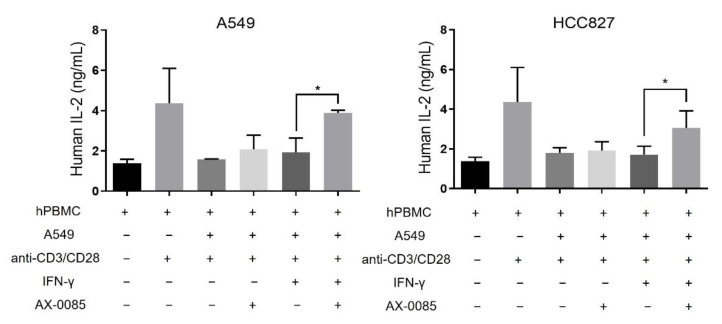
AX-0085 reverses IFN-γ-induced inhibition of T cell activation. Graphs show the results from ELISA assays for secreted IL-2 from PBMC. Treatment with anti-CD3/CD28 antibodies activates IL-2 secretion from PBMC. LUAD cells sequentially treated with IFN-γ and AX-0085 were mixed with activated PBMC, and IL-2 secretion was measured (see Materials and methods section). Reversion of inhibition of IL-2 secretion by LUAD cells or IFN-γ by AX-0085 is shown to be statistically significant. Data are mean ± SEM of three independent experiments (* *p*-value of <0.05 from Student’s *t*-test).

We proceeded to test the effect of AX-0085 on lymphocyte activation again using the two LUAD cell lines (Figure 5). Stimulation of PBMC with anti-CD3 and anti-CD28 antibodies led to increased production of IL-2. This activation was effectively abrogated when PBMC were co-cultured with A549 or HCC827 cells at 1:1 ratio. Importantly, when IFN-γ-treated LUAD cells were further treated with AX-0085, the levels of secreted IL-2 were significantly restored. This is consistent with IFN-γ mediated PD-L1 induction being inhibited by AX-0085, thereby allowing partial activation of T cells and consequent secretion of IL-2 (Figure 5).

### 3.5. Inhibitory Effect of AX-0085 on Tumor Growth

**Figure 6:**
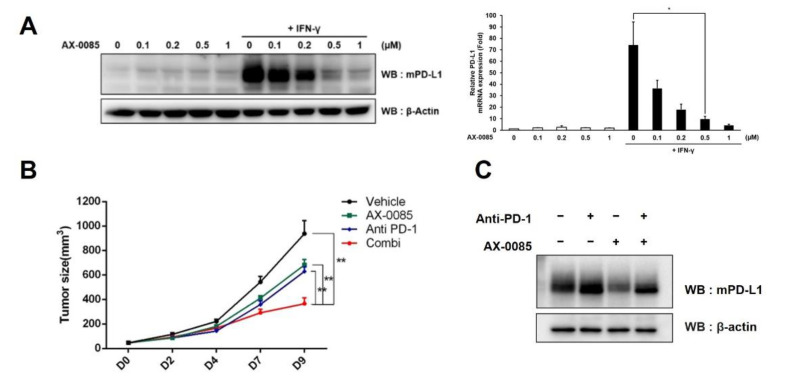
AX-0085 enhances efficacy of anti-PD-1 antibody in vivo. (**A**) Immunoblot showing the inhibition of IFN-γ-induced PD-L1 up-regulation by AX-0085 in a concentration-dependent manner in LLC cells. β–actin was used as the loading control. The concentration of AX-0085 is indicated. Graph shows results from the quantitative real time RT-PCR. Data are mean ± SEM of three independent experiments. Statistical significance is indicated (* *p*-value of <0.05, from Student’s *t*-test). (**B**) Graph showing growth of xenograft tumors (*n* = 5; ** *p*-value of <0.01 from unpaired two-tailed *t*-test). ‘Combi’ indicates combinatorial treatment of AX-0085 and anti-PD-1 antibody. (**C**) Immunoblot showing the inhibition of PD-L1 expression in tumors by AX-0085 with or without anti-PD-1 antibody. β–actin was used as the loading control.

In order to test the efficacy of AX-0085 in vivo, we resorted to a syngeneic mouse model based on utilization of Lewis Lung Carcinoma (LLC) cells and C57BL/6 mice. First, we confirmed that LLC cells up-regulates PD-L1 expression in response to IFN-γ treatment and that AX-0085 down-regulates the induction of PD-L1 (Figure 6A). As in the case of human LUAD cells, inhibition of PD-L1 was shown to involve transcriptional inhibition, as seen by the down-regulation of the mRNA level (Figure 6A). LLC cells were injected to mice 10 days prior to the initiation of treatments, and mice were subsequently treated with (i) vehicle and IgG control, (ii) AX-0085 and IgG control, (iii) vehicle and Anti-PD-1 antibody, or (iv) AX-0085 and Anti-PD-1 antibody. AX-0085 was shown to have anti-tumor activity on its own as daily application at 10 mg/kg dosage inhibited tumor growth significantly. Similarly, anti-PD-1 antibody also had inhibitory effect on tumor growth, as has been previously reported [24,25]. Importantly, we saw a significant decrease in tumor size in mice treated with the combination of AX-0085 and anti-PD-1 antibody compared to those treated with either AX-0085 or anti-PD-1 antibody alone or with neither of the reagents (Figure 6B). We also examined the levels of PD-L1 using the protein extracted from tumor samples (Figure 6C). Treatment with anti-PD-1 antibody led to up-regulation of PD-L1 as has been reported previously, but the addition of AX-0085 visibly decreased PD-L1 expression [24,26,27]. This provides a mechanistic basis for the synergistic effect between AX-0085 and anti-PD-1 antibody in reducing the tumor burden.

## 4. Discussion

Immune checkpoint inhibition is most notable for its durable effect on a significant minority of cancer patients. Why it does not work on the majority is, however, still not clear, and this represents one of the major challenges for the future of cancer immunotherapy [10]. One effort is oriented toward finding good prognostic indicators of the outcome of the immunotherapy [12,28,29]. Albeit limited in predictive power, the expression level of PD-L1 is used as an important indicator for the outcome of the treatment for LUAD, including NSCLC [7,14]. This reflects the fact that tumors with high PD-L1 expression likely rely on its inhibitory effect on T cell activation to evade cytotoxic immune reaction [15,16]. Tumor mutational burden is regarded as another indicator based on that the number of neoantigens expressed in tumor cells would be proportionately greater with higher mutational burden [12,28]. Genomic and transcriptomic analyses to identify polymorphisms and/or differentially expressed genes showing correlation with the treatment outcome are also being actively pursued [28,29].

Another clinically relevant point is that even those patients without durable effects often show short-term or partial benefits. Therefore, combinatorial therapy with other partially efficacious treatments could have additive or synergistic effects [17,18]. Traditional chemotherapy, radiation therapy, and targeted therapy for specific mutated genes such as *EGFR* have been used in combination with immunotherapy [30]. An interesting pursuit is developing therapeutic agents targeting the expression of *PD-L1*. Kasikara and coworkers showed that a pan-TAM (Tyro3, Axl, and Mertk) tyrosine kinase inhibitor enhanced the efficacy of anti-PD-1 antibody treatment in a murine model of triple negative breast cancer [24]. Similarly, Wu and coworkers isolated Regorafenib from a kinase inhibitor library based on the inhibition of IFN-γ-induced *PD-L1* expression [31]. In a murine model of melanoma, the combinatorial application of Regorafenib and anti-PD-1 antibody showed a higher efficacy than the individual application of either reagent. We report in this study that AX-0085 represents another novel kinase inhibitor with a similar activity enhancing the effect of anti-PD-1 antibody. We demonstrate that AX-0085 inhibits JAK2/STAT1 phosphorylation and STAT1 nuclear localization, thereby effectively blocking the IFN-γ-induced up-regulation of PD-L1 expression. Its inhibitory effect of IFN-γ signaling appears to be highly specific and strong, as indicated by transcriptomic analyses. Consistent with inhibition at the stage of JAK2/STAT1 activation, which represents an upstream point of IFN-γ signaling pathway, AX-0085 broadly reversed the expression pattern induced by IFN-γ. In fact, most of the genes belonging to the top GO term groups from IFN-γ treatment showed reversion toward basal gene expression levels. Importantly, the efficacy of AX-0085 as a combinatorial agent when used with anti-PD-1 antibody is also demonstrated using Lewis Lung Carcinoma cells and the syngeneic C57BL/6 mice. The results raise the possibility of further developing this agent or its derivatives as potential supplement in cancer immunotherapy for LUAD. Additionally, as induction *PD-L1* expression by IFN-γ is a phenomenon seen in a wide variety of somatic cells, it would be of interest to test the inhibition of IFN-γ signaling in cells of other cancer types and the combinatorial effect of AX-0085 using syngeneic mouse models of other types of solid tumors.

## Data Availability

The RNAseq data have been deposited in the Gene Expression Omnibus (GEO) database [GEO: GSE185306].

## Supplementary Materials

The following are available online at https://www.mdpi.com/article/10.3390/cells11010019/s1, File S1: Results from densitometric analyses; File S2: DEGs in up-regulated GO term groups; File S3: DEGs in down-regulated GO term groups.
